# Cardiorenal Syndrome: A Literature Review

**DOI:** 10.7759/cureus.41252

**Published:** 2023-07-01

**Authors:** Abimbola O Ajibowo, Okelue E Okobi, Erhieyovbe Emore, Elizabeth Soladoye, Cherechi G Sike, Victor A Odoma, Ibrahim O Bakare, Olasunkanmi A Kolawole, Adebola Afolayan, Emeka Okobi, Chinyereadaeze Chukwu

**Affiliations:** 1 Internal Medicine, Lugansk Medical University, Lugansk, UKR; 2 Family Medicine, Medficient Health Systems, Laurel, USA; 3 Family Medicine, Lakeside Medical Center, Belle Glade, USA; 4 Medicine and Surgery, University of Lagos, Lagos, NGA; 5 Internal Medicine, Piedmont Athens Regional, Athens, USA; 6 General Practice, Windsor University School of Medicine, Cayon, KNA; 7 Cardiology/Oncology, Indiana University (IU) Health, Bloomington, USA; 8 Internal Medicine, University of Texas Southwestern Medical Center, Dallas, USA; 9 Internal Medicine, University of Texas School of Public Health, Texas, USA; 10 Internal Medicine, Triboro Center for Nursing and Rehabilitation, New York City, USA; 11 Dentistry, Ahmadu Bello University Teaching Hospital Zaria, Abuja, NGA; 12 Family Medicine, University of Calgary, Calgary, CAN

**Keywords:** cardio-renal, cardio-renal cascade, cardiorenal syndrome review, cardiorenal syndrome types, cardiorenal syndrome

## Abstract

Cardiorenal syndrome (CRS) is a condition characterized by the intricate two-way relationship between the heart and kidneys, which can lead to acute or chronic dysfunction in these organs. The interplay between cardiorenal connectors and both hemodynamic and non-hemodynamic factors is crucial to understanding this syndrome. The clinical importance of these interactions is evident in the changes observed in hemodynamic factors, neurohormonal markers, and inflammatory processes. Identifying and understanding biomarkers associated with CRS is valuable for early detection and enabling intervention before significant organ dysfunction occurs. This comprehensive review focuses on the clinical significance of biomarkers in the diagnosis, prognosis, and management of CRS. Finally, it highlights the necessity for further advancements in managing this condition.

## Introduction and background

The ‘heart and kidney interaction “coinage was first established in 2004, and over the years, studies have shown cardiac and renal diseases to be common. These diseases have been shown to coexist and significantly increase the mortality, morbidity, complexity, and cost of health care [1]. The heart and kidney possess a bidirectional relationship, which means both organs share physiological and pathological conditions. Subsequently, the heart is reliant on the kidney-regulated homeostasis even as the renal function is increasingly reliant on the blood perfusion function regulated by neurohormonal, hemodynamic, and inflammatory mechanisms [2].

Cardiorenal syndrome (CRS) is a known pathophysiological kidney and heart condition. Either the severe or acute malfunctioning of the organ could lead to the severe or chronic dysfunction of the other organ. The medical condition mainly occurs when an acute or severe dysfunction in either of the organs leads to severe or acute dysfunction of the other organ. There is a prevalence in the overlap of heart and kidney diseases, hence a substantial increase in mortality, morbidity, complexity, and care expense [3,4]. In 2004, the National Heart, Lung, and Blood Institute Working Group in 2004 evaluated the interaction between the heart and kidney. From this evaluation, they defined cardiorenal syndrome (CRS) as the result of interactions between the kidneys and other circulatory compartments that increase the circulating volume, thus worsening the symptoms of heart failure (HF) and disease progression [5]. Cardiorenal syndrome refers to the closer correlation existing between cardiovascular and renal diseases, along with the probability of the interchanging effects that establish their development [6-7,1-78].

Aim of the study

This review aims to provide a comprehensive and up-to-date understanding of the intricate interplay between the heart and kidneys in the context of this multi-organ disease known as cardiorenal syndrome. In pursuit of this objective, the study intends to accomplish several goals. First, it seeks to review the historical background and foundational knowledge surrounding the cardiorenal link. Second, it aims to highlight significant milestones in the comprehension of the relationship between the kidney and heart. Furthermore, the review endeavors to describe the pathology of the cardiorenal syndrome and identify the underlying mechanisms that contribute to its development and progression. Moreover, it explores the classification and subcategories of cardiorenal syndrome. Lastly, the review summarizes the therapeutic options available for managing this syndrome. By achieving these objectives, this paper aspires to contribute to the existing body of knowledge on the cardiorenal syndrome, offering valuable insights for researchers, healthcare professionals, and policymakers involved in the management and treatment of this condition.

Methodology

**Figure 1:**
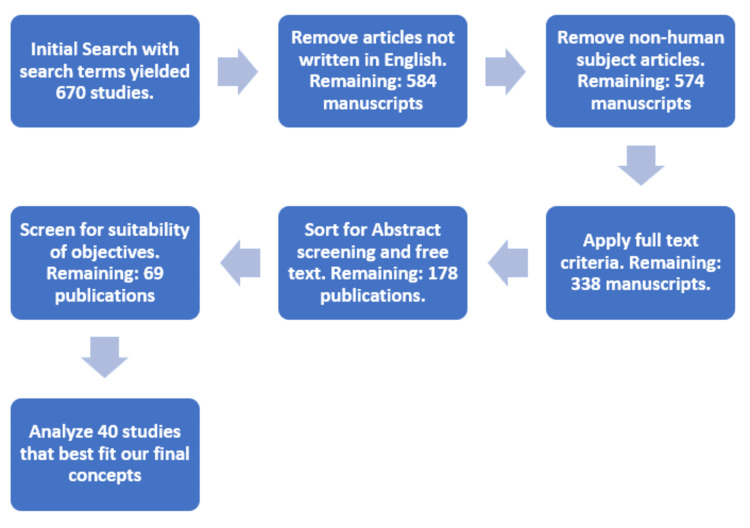
Flow chart of selected studies with criteria

We deployed the journal repository database review approach. Using the Boolean terms “AND’ and “OR,’ our literature search was conducted on PubMed using the keywords 'Cardiorenal syndrome,' 'Cardiorenal syndrome types,' and 'Cardiorenal syndrome review.' Initially, our search yielded a total of 670 articles. To ensure relevance and up-to-date information, we applied inclusion criteria based on the objective of our article's conceptualization plan. Specifically, we focused on articles written in English and focused on human subjects. We also limited our selection to articles with free full texts available, excluding preprints. After applying our inclusion and exclusion criteria, we meticulously reviewed and analyzed 40 publications that met our criteria (See Figure 1 and Table 1 below for included studies).

**Table 1:** Table of selected studies

Selected studies			
Ronco C, et al. [1]. Goffredo G, et al. [2]. Forman DE, et al. [3]. Heywood JT, et al. [4]. House AA, et al. [5]. Ronco C, Haapio M, et al. [6]. Gnanaraj J, et al. [9]. Ronco C, Cicoira M, et al. [10]. Cruz DN, et al. [11]. Bagshaw SM, et al. [12].	Scabbia EV, et al. [14]. Sarnak MJ, et al. [16]. Damman K, et al. [24]. Warnock DG, et al. [28]. McAlister FA, et al [30]. Lacoviello M, et al. [32]. Newman DJ, et al. [33]. Pinsino A, et al. [34]. Singh D, et al. [35]. Nozawa Y, et al. [36].	Comper WD, et al. [38]. Devuyst O, et al. [43]. Lacoviello M, et al. [44]. deBoer RA, et al. [45]. Grande D, et al. [46]. Rebholz CM,et al. [47]. Mishra J, et al. [50]. Lekawanvijit S, et al. [56]. Savira F, et al. [59]. Lin CJ, et al. [61].	Testani JM, et al. [63]. Collins SP, et al. [67]. Dormans TP, et al. [68]. Yusuf S, et al. [69]. Go AS, et al. [70]. Heywood JT, et al. [71]. Pinsino A, et al. [74]. Ronco C, et al. [75]. Vergaro G, et al. [77]. Kirklin JK, et al. [78].

## Review

**Figure 2:**
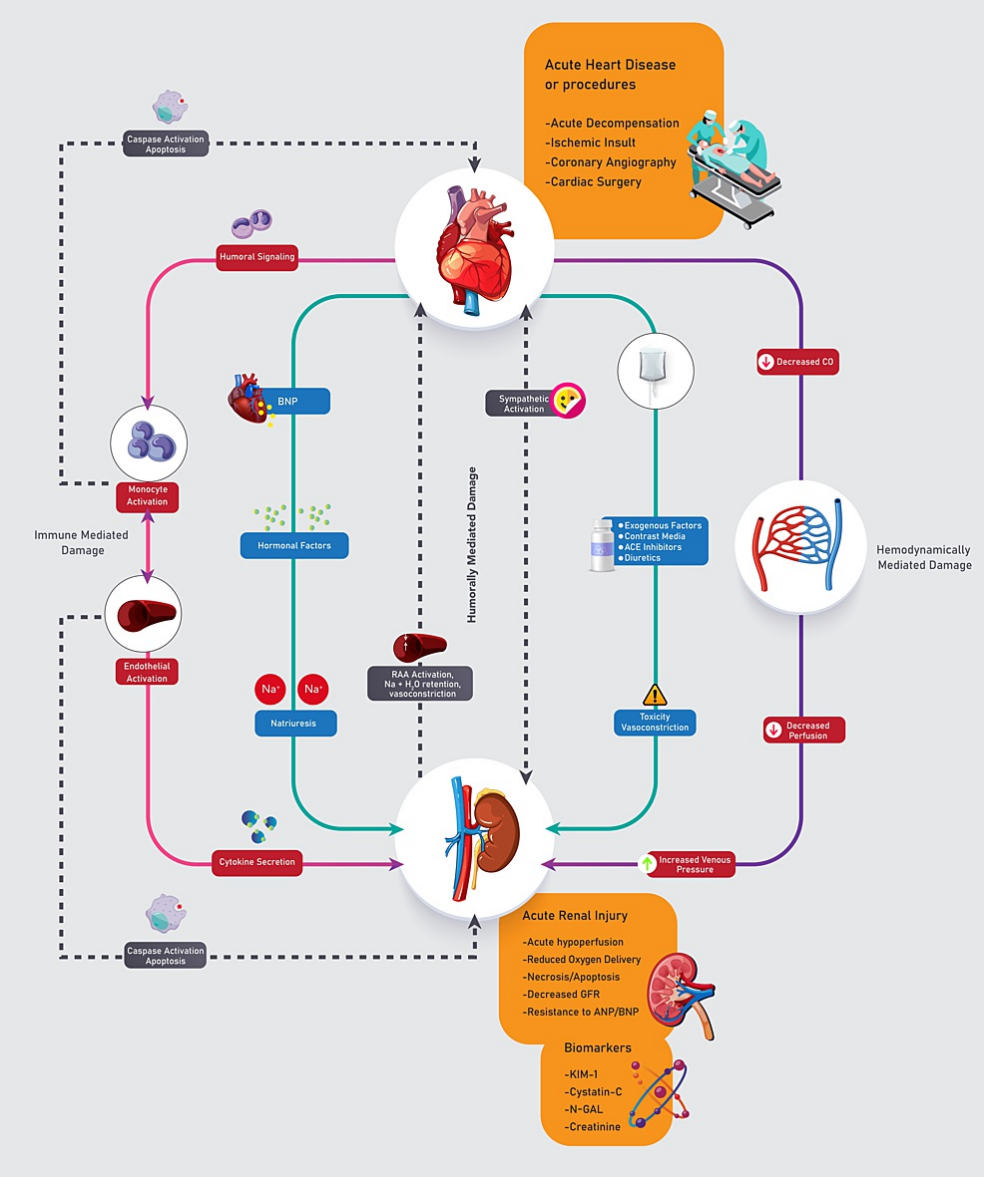
Cardiorenal syndrome flow chart This illustrative diagram is an original flow chart designed by the authors of this manuscript.

The cardiorenal syndrome is defined to include five varying sub-types (I-V); these subtypes are categorized with respect to both primary and secondary pathology, the time frame for the incidence, and concurrent renal and cardiac co-dysfunction, which is consequent to the systemic disease [5-8]. The underlying pathologic mechanisms responsible for this heart and kidney interaction cannot be traced using this sub-type categorization; however, it represents the primary dysfunction that was a precursor to CRS. From these subtypes, type V cannot be said to fit the generally accepted CRS description, and heart and kidney dysfunction in sub-type V results from a culmination of various systemic diseases [9]. However, CRS incidence is dependent on subtypes. Acute Kidney Injury (AKI) has been observed to occur in approximately 25%-33% of cases involving acute decompensated heart failure (ADHF). Studies have affirmed this as a risk factor related to hospital admission, the requirement for renal replacement, mortality, readmission, and an increment in stroke risk [9,10]. Concurrently, it has been reported that in 60% of instances involving acute decompensated heart failure (ADHF), acute kidney injury (AKI) can be seen as an exacerbation of a previously diagnosed chronic kidney disease (CKD). However, in chronic heart failure (HF), chronic kidney disease has been reported to be a co-morbidity in 26% to 63% of patients [9,11]. A graphical representation of cardiorenal syndrome and all its identifiable contributing components can be seen in Figure 2.

Cardiorenal syndrome and its sub-types

In continuation of the above-established definition and understanding of cardiorenal syndrome (CRS), it can be clearly stated that there is an establishment of five (5) basic subtypes of CRS. Table 2 shows the diverse CRS subtypes alongside their epidemiology and impacts on clinical and healthcare outcomes [9]. 

**Table 2:** Classification, etiology, and impact on clinical outcomes of cardiorenal syndrome This table is an original creation from several pieces of literature [1-4,9-16]

Subtypes of cardiorenal syndrome	Etiology	Impact on the clinical outcome of the patient
Type I (Acute cardiorenal syndrome/Acute cardiogenic renal failure)	Seen in patients with acute decompensated heart failure, myocardial infarction, or other acute cardiac conditions.	Acute kidney injury in acute cardiac dysfunction is linked to worse outcomes.
Type II (Chronic cardiorenal syndrome)	Seen in patients with chronic heart failure, where the progressive decline in cardiac function contributes to developing or worsening chronic kidney disease.	Has a poorer prognosis due to the combined effects of heart failure and chronic kidney disease.
Type III (Acute reno-cardial syndrome)	Occurs in acute kidney injury, where renal dysfunction contributes to developing or exacerbating cardiac abnormalities.	Associated with adverse cardiac outcomes, such as arrhythmias.
Type IV (Chronic reno-cardial syndrome)	Frequently observed in patients with end-stage renal disease. The renal impairment, fluid overload, and electrolyte imbalances associated with CKD can lead to cardiovascular complications.	Patients have a higher risk of cardiovascular events.
Type V (Secondary cardiorenal syndrome)	It entails various systemic conditions, such as sepsis, autoimmune diseases, and liver disease, which can simultaneously affect both the heart and kidneys (Ahmed et al., 2007).	Prognosis depends on the underlying systemic disorder.

CRS type I (acute cardiorenal syndrome)

Approximately 25% to 35% of patients admitted with acute decompensated heart failure (ADHF) had acute impairment of cardiac function, leading to renal dysfunction; this invariably depends on the employed criteria, in addition to having critical implications with regard to prognosis, diagnosis, and management [1,9]. A prerequisite for the acute cardiorenal syndrome is when the severe deterioration of the various cardiac functions is likely to result in “Acute Kidney Injury (AKI)” and/or Acute Renal Failure (ARF). Certain attributes of acute cardiac conditions enhance the deterioration of renal functions in a way comparable to cardiorenal syndrome type 1 development, and these stated conditions include acute coronary syndrome, cardiogenic shock, acute decompensated heart failure (ADHF), and low flow syndrome following cardiac surgery [4]. Moreover, acute renal failure after surgery (cardiac and non-cardiac) is a typical clinical condition associated with CRS type I. Also, type 1 CRS may occur without the use of contrast media and has been acknowledged to occur in approximately 25% of hospitalized ADHF patients. Among such patients, CKD is common and adds to AKI in 60% of studied cases, even though AKI is one of the independent mortality risk factors in ADHF patients [5]. Severe renal function impairment, as indicated by an increment of baseline serum creatinine above 0.3 mg/dl, takes place in variable proportions in 27% to 40% of individuals with severe coronary syndrome and acute heart failure [5]. Studies have indicated that cardiorenal syndrome type 1 development may lead to considerable deterioration with regard to prolonged hospital admission, mortality, and morbidity [12-15].

CRS type II (chronic cardiorenal syndrome)

The chronic cardiorenal syndrome is common and usually occurs when renal failure develops with coexistent chronic cardiac dysfunction. Severe cardiac impairment entails several divergent heart conditions, including atrial fibrillation, chronic ischemic heart disease, chronic heart failure (CHF), constrictive pericarditis, and congenital cyanotic heart disease. An estimate of 63% with chronic congestive heart failure is known to present a picture of chronic heart renal failure ranging from stages 3-5 [5,14].

Due to the common concomitance of severe kidney disease and chronic cardiac impairment, it is difficult to determine which conditions are pre-existing; thus, an accurate anamnestic reconstruction is paramount. Furthermore, the concomitance of the renal and cardiac conditions tends to worsen the prognosis in relation to the increased risk of morbidity and mortality [4,13-15]. Therefore, it may be concluded that renal failure is one of the negative prognostic factors with regard to the independent evaluation of the heart failure framework’s evolution.

CRS type III (acute reno-cardial syndrome)

CRS type III is less studied; its prevalence is unknown and most identifiable. CRS type III occurs when acute renal function (ARF) deterioration results in the damaging and development of acute cardiac dysfunction; the CRS type III condition boundaries have still remained unidentified, and this is because identification still grossly relies on occasional and non-systematic reports [9,14]. Certain typical clinical conditions are associated with CRS type III, including (1) drug-induced acute renal disease, (2) rhabdomyolysis, and (3) acute nephritic syndromes.

Additionally, CRS type III is an acute renal failure resulting from medial contrast, particularly in instances where it is not determined by diagnostic tests that evaluate other pre-existing heart diseases/conditions. Without diagnostic testing, it would be difficult to diagnose or identify CRS type III, given its close resemblance to CRS type I. Such conditions, including acute kidney injury (AKI), develop alongside acute decompensation, acute coronary syndrome, congestive heart failure (CHF), and arrhythmias, among others [1,9].

CRS type IV (chronic reno-cardial syndrome)

CRS type IV possesses the condition where primary chronic renal failure (CRF) and primary chronic kidney disease could contribute to the reduction of cardiac function and help the deterioration of cardiac function, such as left ventricular diastolic dysfunction, left ventricular hypertrophy, and cardiac remodeling. This deterioration increases the risk of acute cardiovascular events like stroke, acute heart failure, and myocardial infarction [9,14].

Patients with chronic renal failure have a mortality rate 10 to 20 times higher than in a comparable population by age and sex without Idiopathic Restrictive Cardiomyopathy (IRC). Observational and population studies have culled documentation of a rising trend in morbidity and mortality from cardiovascular disease with the passage of worsening stages of renal function ranging from stage 1 to 3 [6,14-17]. Chronic kidney disease is renowned as an independent predictor of cardiovascular disease, which is also independent of age and conventional risk factors [9,18]. A systematic review of 13 studies reporting cardiovascular and all-cause mortality in non-dialysis-dependent chronic kidney disease (CKD) patients observed that the increased risk for all-cause mortality was influenced by cardiovascular deaths having a 58% death ratio [9,19].

CRS type V (secondary cardio-renal syndrome)

CRS type V is associated with acute or chronic systemic illness, which is known to induce cardiac and kidney injury and/or dysfunction. Some common conditions lead to the dysfunction of both kidney and heart; these include amyloidosis, heroin, cocaine, sepsis, chemotherapeutic drugs, hepatitis B and C, systemic lupus erythematosus, diabetes mellitus, and sarcoidosis, to name a few [9,14]. Aggravating co-existing conditions like diabetes and/or hypertension are likely to increase the impairment severity of both organs (heart and kidney); also, bilateral renal artery stenosis could manifest as recurrent episodes of flash pulmonary edema [9]. The physiopathological attributes of the CRS type V condition have yet to be properly defined; however, it has its own epidemiological logic [14].

Pathophysiology of cardiorenal syndromes

Cardiorenal syndromes cannot be talked about without diving into their pathophysiology. CRS presents a complex pathophysiology that includes dysfunction of the neurohormonal system, abnormal endothelial activation, and the release of pro-inflammatory cytokines. Research has shown that these pathophysiological mechanisms work simultaneously and sequentially. This, in turn, leads to cardiac and renal fibrosis and, ultimately, dysfunction [14].

Associated pathophysiology

Abnormal endothelial activation: The volume overload that results from renal or cardiac dysfunction often leads to the endothelial cells experiencing a circumferential stretch. Thus, the resultant biochemical stress stimulates them alongside such cells, and as a result, they alter their varied synthetic profiles from the original quiescent state into an increasingly activated state. Also, regarding heart failure, the mechanism acts through the perpetuation of vicious circles that waver in the state of acute renal hypoxia, oxidative stress, and inflammation that alter renal and cardiac structure and functions. [14,20]. Accordingly, an increment in the pro-inflammatory cytokine concentrations, similar to interleukin 6 (IL-6) and tumor necrosis factor, occurs, thereby impairing both renal and myocardial functions, which, in turn, leads to the speeding up of the progression of heart failure (HF) [14,21].

Neurohormonal Dysfunction

CRS type I and type II portray venous congestion along with considerable reductions in cardiac output as a result of cardiac dysfunction, leading to a reduction in the glomerular filtration rate (GFR). The activation of the renin-angiotensin-aldosterone system (RAAS) also occurs, which further stimulates the non-osmotic arginine-vasopressin release alongside various neuroendocrine hormones that include endothelin, which, in turn, leads to renal injury [14]. Various recent clinical studies have indicated an increment in the levels of plasma catecholamine among renal dysfunction patients, which also portrays sympathetic hyperactivity in both CRS type III and CRS type IV [14]. The affected or dysfunctional kidneys act by relaying afferent signals to the patient’s central nervous system, resulting in an increment in sympathetic nerve discharge in addition to contributing to the development of hypertension, deterioration of renal functions, and cardiac injuries. Additionally, RAAS activation has been observed to enhance the levels of angiotensin II, leading to the enhancement of aldosterone secretion, which further leads to water and sodium retention. Studies have additionally disclosed that angiotensin II has a direct effect on renal tubular cells and cardiomyocytes and is also known to promote fibrosis, cellular hypertrophy, and apoptosis [14,22].

Endotoxemia, Inflammation, and Infection

Myocyte and renal dysfunction may be exacerbated by intestinal congestion and hypoperfusion from renal and cardiac dysfunction that leads to intestinal translocation of the bacterial endotoxin into the systemic circulation. The outcome entails the activation of circulating immune cells through the release of cytokines similar to tumor necrosis factor-alpha, interleukin-1 (IL-1), and interleukin-6 (IL-6) [14,23].

The Venous Congestion Role

In medicine, it has been widely observed that worsening renal function is likely to result in heart failure, mainly due to hypoperfusion. Even though hypoperfusion mainly results in renal injuries, it is not considered the single mechanism through which heart failure may lead to renal dysfunction. A study conducted by Mullens et al. has disclosed that low-output decompensated heart failure (HF) venous congestion patients, as indicated by an increment in the central venous pressure (CVP) during hospital admission, and inadequate CVP reduction during hospital admission is regarded as the sturdy hemodynamic determinants with regard to the worsening renal function development [14]. There is also an inverse correlation between GFR and CVP with regard to congestive heart failure (CHF) [14,24]. The rise in CVP leads to an elevation in renal venous pressure, which, in turn, increases renal interstitial hydrostatic pressure. In cases where the interstitial hydrostatic pressure surpasses the tubular hydrostatic pressure, the tubules will likely collapse, and the net ultrafiltration pressure will considerably reduce, resulting in renal dysfunction [14,25].

Predisposing factors contributing to CRS

As stated before, there is a continuous rise in the number of people having cardio-renal syndrome all over the globe, and to better understand and treat the condition. To reduce its prevalence, there is a need to examine, analyze, and proffer solutions to the cardio-renal syndrome. To arrive at this, there is a need to study predisposing factors that enhance CRS; a few of these predisposing factors are listed below.

Anemia and Nutritional Deficiencies

Having anemia, cachexia, and nutritional deficiencies usually results in the elevation of tumor necrosis factor-alpha along with different pro-inflammatory cytokines known to be a result of chronic kidney disease and heart failure (HF). Eventually, this contributes to further damage and fibrosis in the other organ [3,14].

Obesity

Research states that obesity-related glomerulopathy is a condition of hyperfiltration in obese individuals that don’t have diabetes mellitus (DM) and ultimately leads to chronic kidney disease (CKD) and cardiorenal syndrome (CRS), especially in types II and IV [14,26]. Additionally, in frank diabetes mellitus absence, the cardiometabolic syndrome has been linked to a nearly seven-fold increment in the cardiorenal syndrome type I risk, particularly in the clinical context host [14,27]. Further, the adipocytes have been acknowledged to secrete IL-6 along with tumor necrosis factor-alpha, which have been implicated in the progression of cardiac and renal diseases [14].

Hypertension

It is known that elevated blood pressure results in direct renal and cardiac injuries in addition to reflecting the increment in the activation of the sympathetic neurohumoral. This has additionally been linked to the increase in incidence and deterioration of renal failure, particularly in patients with decompensated congestive heart failure.

Diabetes

In many ways, diabetes has been acknowledged to aid in glomerular damage and dysfunction, the functional filtration units’ eventual loss, and the development of chronic kidney disease. Moreover, mesangial, podocyte and endothelial injuries in both diabetes mellitus and hypertension often result in the secretion of extra quantities of albumin within the Bowman's space. Accordingly, this results in the proximal tubular cells acquiring increased reabsorption workloads [14]. The phenomenon has been shown to lead to renal tubular cell apoptosis, an increment in nephron loss rates, and the progression of kidney disease. Severe kidney injury in various settings is associated with albuminuria and gross proteinuria.

Cardiorenal syndrome clinical biomarkers

Numerous biomarkers have been researched over the years to better evaluate the severity of renal dysfunction and accurately pinpoint early risks of cardiorenal syndrome progression [2,7]. Different biomarkers have relationships according to the cardiorenal syndrome’s diverse pathophysiological factors. These relationships vary from systemic inflammation to those that reflect tubular and glomerular function and to those related to the end stage of renal disease, like uremic toxins [2]. Each stage has biomarkers attributed to it, and we shall attempt to talk about these biomarkers in detail to arrive at a conclusive understanding of how they affect the diagnosis, prognosis, and treatment of the cardiorenal syndrome.

Biomarkers in kidney and cardiovascular diseases

The past decade has brought about an accurate estimation of glomerular filtration rate (GFR) based on aspects that include the level of serum creatinine, gender/sex, race, age, and weight through various empirical formulas [2]. Renal function is known to be generally assessed using formulas. These formulas estimate the glomerular filtration rate based on serum creatinine levels [2,27-29]. Serum creatinine is the result of skeletal muscle creatine phosphate breakdown. Serum creatinine’s rate of production is known to be relatively constant, while its elimination by the kidney is mainly overseen by glomerular filtration alongside tubular secretion [2,30]. In patients with chronic heart failure, especially with renal functions that are normal or partly normal, the GFR-EPI enables increasingly precise categorization of renal function and improved stratification of risks [2,30].

Creatinine Serum

This is regarded as the cornerstone in diagnosing the presence of chronic kidney syndrome and its progression. It should be noted that its varying limitations are to be considered. Factors like age, race, diet, gender, and body mass could influence creatinine serum levels. Also, cardiac cachexia and muscle wasting could decrease creatinine serum, thus causing an overestimation of eGFRcr, as seen in advanced heart failure [2,31]. Depending on eGFR alone cannot enable the detection of the early presence of pathophysiological conditions that result in renal dysfunction [31,32]. The above-stated limitations are increasingly pertinent in patients with the cardiorenal syndrome, as these demand a precise approximation of renal function. Renal biomarkers have to respond to two key clinical requirements in addition to the GFR: they must enable an enhanced approximation of renal function status along with its worsening. In contrast, they should more accurately detect the predisposing pathophysiological conditions to acute or worsening renal function that could otherwise represent a therapeutic target [2,7].

Cystatin C

This is a cysteine proteinase inhibitor and is regarded as a useful tool to compensate for a few of the limitations associated with estimating GFR regarding creatinine serum levels. Cystatin C is secreted by all nucleated cells and freely filtered by the glomerulus, in addition to being reabsorbed and not secreted within the tubular cells. Moreover, Cystatin C is less reliant on aspects that include age, cachexia, nutritional status, and weight [2,33]. An increasingly precise approximation of the renal functions through Cystatin C during the prediction of the initial postoperative results in advanced heart failure, including the left ventricular assisting device recipients, makes it better compared to creatinine serum [34]. Certain factors influence the rise in Cystatin C levels, including obesity, inflammation, concomitant steroid therapy utilization, and thyroid dysfunction [35,36]. Cystatin C is accurate in stratifying the risk of events in the elderly and patients affected by CVD (coronary artery disease, acute and chronic heart failure). The limitations of using Cystatin C in routine clinical practice are its relatively high cost and its use in the detection of renal dysfunction in instances where only creatinine levels are biased [2,33].

Renal Function Reserve

It is among the potential diagnostic procedures that offer a better evaluation of renal function status. The evaluation of renal function reserve (RFR) might be clinically important, given that it represents the kidney’s ability to enhance GFR and the response of glomerular filtration to stimuli in relation to diverse pathophysiological conditions [2,37,38]. RFR vicariously enhances the residual nephrons’ glomerular filtration rate via GFR maintenance and the lost function. This is attainable as a result of the GFR being capable of remaining within set normal values up to a time when 50% or half of the nephrons have been lost or have failed. RFR testing might proffer an increasingly sensitive means for accurate assessment of the renal system’s functional decline and the kidney’s aptitude to effectively recover from severe injuries [2,37]. To evaluate RFR, present methodologies are not straightforwardly practicable in clinical practice, and this can be attributed to protein loading along with the re-evaluation of the levels of creatinine serum. In instances of a reduction in RFR levels, the kidney is highly prone to becoming increasingly disposed to experiencing worsening renal function.

Microalbuminuria

The evaluation of microalbuminuria provided the necessary parameter that is reflective of existing anomalous renal microcirculation, given that, on normal occasions, urine albumin levels tend to be lower, attributable to the smaller size and negative charge as well as the limited tubular absorption [38]. The existence of inflammation, endothelial dysfunction, elevated glomerular pressure, and atherosclerosis may lead to damage and injuries to the glomerular membrane, which might, in turn, lead to an increment in the secretion of albumin [38,39]. In instances of chronic heart failure, the existence of microalbuminuria might be indicative of the various abnormalities related to renal hemodynamics. That is, albuminuria is considered an indicator of the pathophysiological background that underlies the progression of chronic kidney disease [38,39]. In the assessment of renal dysfunction, albuminuria incorporates information gained from the approximated GFR to enable the improved staging of chronic kidney disease as well as stratified prognosis [29]. The ratio assessment between creatinine and urinary albumin (UACR) is currently based on the severity and existence of albuminuria. Microalbuminuria has been described as being 30 to 300 mg/g UACR, even though macroalbuminuria is > 300 mg/g UACR [39]. Further, even as Microalbuminuria is increasingly common in congenital heart failure, its existence implies an increased possibility of a worse prognosis, independent of estimated GFR and creatinine serum levels [40]. Thus, increased excretion of urine albumin ascertains a higher risk of cardio-renal syndrome.

Tubular Biomarkers

Various studies have indicated that tubular biomarkers can be used to predict the progression of renal dysfunction, especially in CHF patients. As the proximal tubule’s lysosomal protein, N-acetyl beta glucosaminidase (NAG), passes into the patient’s urine in instances of tubular damage or injury, the kidney injury molecule (KIM1), which refers to the transmembrane glycoprotein, can be found in cells following hypoxic tubular injury [2]. KIM and NAG serum has additionally been acknowledged to predict increments in mortality risk in heart failure or congenital heart failure patients that are conducted independently of GFR. Uromodulin, beta-2 microglobulin (B2M), and alpha-1 microglobulin (A1M) have been acknowledged to play vital functions. For instance, A1M, a liver-synthesized plasma protein, has antioxidant properties and is always filtered by the renal glomerulus before complete reabsorption through the renal tubule. Nonetheless, renal tubular injuries are mainly detected in urine samples [41]. B2M, being a non-glycosylated low molecular weight protein, is mainly observed on every nucleated cell surface, and just a smaller proportion of B2M can be found in the urine. B2M’s concentration, nevertheless, increases in instances of injuries to the renal tubules [42]. Consequently, the production of uromodulin protein only occurs in the kidney and can be found in healthy individuals’ urine. Nevertheless, the functions of uromodulin have not been fully established, even though there is consensus among researchers and scientists that its key function entails regulation of ion transport and, as a result, prevents the development of kidney stones, kidney injuries, and kidney infections [43].

Gelatin-3

Apart from microalbuminuria, Gelatin-3 (Gal-3) is another notable biomarker that offers the necessary information regarding the pathophysiological background that underlies and highlights renal dysfunction and the accompanying progression in patients with CVD [32,44]. The key pathophysiological function of Gal-3 is to promote fibrosis, induce fibroblast proliferation, and activate collagen deposition in the extracellular matrices after secretion and release by the macrophages. [31]. Through these functions, Gal-3 not only promotes cardiac remodeling but also enables the progression of heart failure, in addition to playing an active role in renal fibrosis and dysfunction, a factor disclosed by various experimental studies [45,46]. The levels of Gal-3 in humans have direct correlations with the GFR. Moreover, higher levels of Gal-3 and plasma gelatin-3 have been associated with an elevated risk of CKD development [47-49]. Based on several preliminary data acquired, different studies have disclosed that, in the near future, Gal-3 will be considered a vital marker with regard to the progression of cardiorenal syndrome and will additionally be a therapeutic target [48-64].

Neutrophil Gelatinase-Associated Lipocalin (NGAL)

NAG and KIM-1: NGAL is a lipocalin family protein that is expressed by neutrophils along with different epithelial cells. As a minute protein, NGAL is filtered by the glomerulus and subsequently absorbed by the proximal part of the tubule. Moreover, under normal circumstances, NGAL production occurs in the kidney and other organs. The concentration of NGAL in the urine and blood is also low [49]. In cases of tubular damage resulting in the inability of NAGL to be fully reabsorbed, the urinary levels are prone to increase and precede the rise in serum creatinine by less than 24 hours, as another notable tubular marker [50]. Studies have disclosed that NGAL is a key biomarker for renal dysfunction worsening that leads to negative clinical outcomes [51-54]. Nevertheless, the significance of such with regard to clinical practice remains undetermined. Additionally, recent findings indicate that the levels of NGAL do not present increased precision in relation to levels of creatinine in the detection of a poorer prognosis [24,51]. The use of NGAL has several limitations that include the non-univocal sampling frequency and the influence on NGAL levels of different confounding conditions that include sepsis, inflammation, cancer, anemia, hypertension, anemia, and hypoxemia [2,49,65-70].

Fatty Acid-Binding Proteins (FABPs)

These are proteins that bind free fatty acids. The expression of the liver-specific FABP (FABP-1) and the heart-specific FABP (FABP-3) occurs within the proximal and distal tubules, respectively. In this regard, AKI and ischemic tubular risk have been directly linked to increments in urinary FABP-1 and FABP-3 levels, and in patients with chronic heart failure, elevated FABP-3 levels have been associated with elevated cardiovascular occurrence [2,52].

Tissue Inhibitor of Metalloproteinase 2 (TIMP-2) and Insulin-Like Growth Factor Binding Protein 7 (IGFBP7) 

These are novel biomarkers whose markers are a result of insults such as ultraviolet radiation, drugs, inflammation, ischemia, oxidative stress, and toxins [2,53,54]. Further, the two markers play active roles with regard to the G cell-cycle arrest process during the initial phases of cell injury, which prevent cell division in instances of DNA damage, up to such a time that the damage gets repaired before the cell’s demise and senescence [2,54]. In this regard, the biomarkers might act as alarm proteins in the event of tubular damage. The precision of both IGFBP7 and TIMP-2 with regard to predicting AKI occurrence is greater in comparison to Cystatin C, KIM-1, L-FABP, NGAL, and IL-18 [55,56].

P-Cresyl Sulfate (PCS), Indoxyl Sulfate (IS), and Trimethylamine N-Oxide (TMAO)

The increase in the levels of uremic toxins is attributable to the progressive inability to effectively eliminate substances arising from human metabolism, the symbiont, and the intestinal microbiota [56]. Of the toxins, IS, TMAO, and PCS are the only ones that have adequate evidence. IS and PCS are derived from the degradation of different types of aromatic amino acids, including tryptophan, tyrosine, and phenylalanine, while TMAO is mainly derived from animal-origin product catabolism, including products such as betaine, choline, phosphatidylcholine, and carnitine [57,58]. In CKD patients, IS and PCS have been observed to attain a 100-fold increase in comparison to healthy individuals. Both IS and PCS are affected by oxidative, pro-inflammatory, and pro-fibrotic stress inductions at the cardiovascular and renal levels [57,58]. Additionally, IS and PCS can induce and promote cardiac hypertrophy, which, in turn, favors cardiac dysfunction progression [59]. Therefore, both IS and PCS relevance is mainly supported by their observed association with poor prognosis in patients with renal impairment and CVD. Nonetheless, they are increasingly challenging to remove using traditional dialysis, given their increased protein binding ability [60,61]. Therefore, targeting the toxic solutes offers the necessary therapeutic opportunity to reduce the progression of CRS [56,61].

These above biomarkers and their characteristics can be gleaned and better understood by their different representations, as seen in Table 3 [2].

**Table 3:** Cardiorenal syndrome biomarkers This table is an original creation from several pieces of literature [1-6, 9-16]

Marker	Biological function	Clinical Significance	Proposed Cut-Offs
Cystatin C	It functions as a cysteine protease inhibitor. The kidneys freely filter Cystatin C.	Estimates kidney function.	>1.5 mg/L (In blood)
Galectin 3	Associated with the progression of cardiac dysfunction and renal impairment.	Elevated levels show the progression of cardiac dysfunction and renal impairment.	>13.5ng/ml for GFR >60 >18.1ng/ml for GFR < 60
N-acetyl beta glucosaminidase. (NAG)	Enzyme found primarily in the proximal tubules of the kidneys.	Elevated levels in urine indicate renal tubular injury. It is used to assess the progression of CRS.	>50 microg/L (urine)
Kidney injury molecule 1 (KIM-1)	It is upregulated in response to hypoxic renal injury.	Increased levels suggest worsening kidney and cardiac dysfunction due to subsequent hypoxic tubular injury.	From 10 to 15 mg/ng (urine)
Fatty acids binding proteins (FABPs)	A family of small cytoplasmic proteins that transport and metabolize fatty acids.	Increased levels are associated with decreased renal function and an increased risk of adverse outcomes.	>15 microg/g Cr (urine)
P-Cresyl Sulfate (PCS), Indoxyl Sulfate (IS)	Uremic toxins of intestinal derivation from the degradation of aromatic amino acids.	Associated with a worse prognosis in patients with cardiovascular disease and renal impairment.	More than 100 times higher than in healthy subjects
Neutrophil gelatinase-associated lipocalin (NGAL)	Freely filtered through the glomerulus and completely reabsorbed in the proximal part of the tubule.	In acute decompensated HF patients, NGAL rises.	>50 microg/L (urine)

Managing cardiorenal syndrome

Cardiorenal syndrome, a major concern, must be managed properly to reduce mortality and morbidity rates. To achieve this, several general concepts are involved in managing CRS. The most reliable way to achieve this management effectively involves a multidisciplinary, multidimensional strategic, and systematic approach [70-77].

Prevention

There is no definitive form or therapy for directly treating any of the above-stated cardiorenal syndromes (types I-V). Hence the best and most effective way to go about it involves prevention. Once a patient is diagnosed with either heart or kidney dysfunction, there is a high risk for CRS development. However, tackling the conventional risk factors for cardiovascular disease, including hypertension, diabetes, and stopping smoking, are some of the measures that are effective in addressing the problem of cardiorenal syndrome. Patients who suffer from CVD and develop CHF adversely affect prognosis [9,62]. In HF patients with reduced ejection fraction, the addition of angiotensin-converting enzyme (ACE) inhibitors to traditional treatment aids in the reduction of CHF decompensation incidences and HF-associated hospitalization cases. Research shows that renal function worsening in patients being treated for decompensated CHF might be associated with over-diuresis and the increased usage of calcium channel blockers, which might have an indirect correlation to the use of RAAS inhibitors [62]. Also, using drugs that directly cause renal injuries, including various contrast agents and anti-inflammatory medication, should be avoided, particularly in patients with or at elevated risk of developing CHF [9,62].

Congestion Treatment

Congestion plays a key role with regard to the different CRS types’ pathogenesis. Thus, it might result in an increasingly vicious cycle of organ dysfunction via multiple pathways, leading to worsening cardiac and renal prognostic significance despite the worsening of renal parameters [63]. Congestion treatment mainly entails a restriction on the intake of salt and diuretics. The diuretic dosage is mainly based on aspects that include renal function and the diuretics’ pharmacokinetic attributes. Applying once-daily dosing of loop diuretics like furosemide could result in a rebound increase in sodium absorption, making twice-a-day loop diuretics advisable. In instances of resistance, the addition of mineralocorticoid receptor antagonists (MRAs) and thiazides is prone to stimulate diuresis through the decrement of sodium reabsorption within the distal tubule [9,64,65].

Renin-Angiotensin-Aldosterone System (RAAS)

The stimulation of RAAS through the retention of excess water and salt in CRS changes both cardiac preload and cardiac afterload. Consequently, this is prone to worsen both cardiac and renal functioning. Blocking the RAAS using ACE inhibitors and angiotensin receptor blockers could help break this cycle, thus preventing further cardiorenal injury. Moreover, SGLT2 inhibitors play an important role in improving cardiovascular and renal outcomes through the simultaneous prevention of blood glucose highs and lows, which, in turn, lead to comparatively smaller effects on the HbA1C values. Using RAAS blockade with ACE inhibitors in CHF would not have adverse prognostic significance despite the worsening renal parameters [9,63]. Spironolactone and eplerenone may inhibit neurohormonal surge, thus preventing cardiac and renal function in CRS. There should be careful monitoring of patients for hyperkalemia when given alone with ACE inhibitors, especially in instances of pre-existent renal dysfunction; such medications may subsequently aid in overcoming loop diuretic resistance whenever employed with regard to hypervolemia [9,65,73-74].

Left Ventricular Assist Devices (LVAD)

The end-stage HF treatment has been made possible by a device known as the revolutionary implantable mechanical circulatory. Apart from being utilized in the provision of support to an individual awaiting heart transplants, LVAD is currently in use as destination therapy in patients considered ineligible for heart transplants [9,65]. Regarding the indications for LVADs, it can be noted that the devices are indicated in HF patients who are inotrope-dependent, with predominant or pure left ventricle dysfunction. Further, in HTx-listed patients incapable of maintaining end-organ perfusion, LVAD is used as the bridge to transplantation. Also, LVADs are indicated for patients with HTx contraindications, which can be reversed through a duration of support while on LVAD. Consequently, as a destination therapy, LVAD is presently considered the best alternative to a heart transplant, even as pre-LVAD implant renal dysfunction is an indicator of the high risk and rate of mortality rate following the implantation of LVAD [9,66,68-70,78]. As such, it is immensely important that patients get referred for LVAD placement for cardiac indications before HF worsens.

## Conclusions

In conclusion, the treatment of cardiorenal syndrome remains a challenge, and it is essential to acknowledge the complex interaction between the heart and the kidney. Implementing preventive measures to modify risk factors is crucial while providing hemodynamic support and employing appropriate pharmacological interventions are key treatment components. Additionally, continued research into novel devices and therapeutic techniques will contribute to improving CRS treatment standards. By focusing on these aspects, healthcare professionals can effectively enhance their ability to manage and treat this complex syndrome.

## References

[1] Ronco C, McCullough PA, Anker SD (2010). Cardiorenal syndromes: an executive summary from the consensus conference of the Acute Dialysis Quality Initiative (ADQI). Contrib Nephrol.

[2] Goffredo G, Barone R, Di Terlizzi V, Correale M, Brunetti ND, Iacoviello M (2021). Biomarkers in cardiorenal syndrome. J Clin Med.

[3] Forman DE, Butler J, Wang Y (2004). Incidence, predictors at admission, and impact of worsening renal function among patients hospitalized with heart failure. J Am Coll Cardiol.

[4] Heywood JT (2004). The cardiorenal syndrome: lessons from the ADHERE database and treatment options. Heart Fail Rev.

[5] House AA, Anand I, Bellomo R (2010). Definition and classification of cardio-renal syndromes: workgroup statements from the 7th ADQI Consensus Conference. Nephrol Dial Transplant.

[6] Ronco C, Haapio M, House AA, Anavekar N, Bellomo R (2008). Cardiorenal syndrome. J Am Coll Cardiol.

[7] Damman K, van Deursen VM, Navis G, Voors AA, van Veldhuisen DJ, Hillege HL (2009). Increased central venous pressure is associated with impaired renal function and mortality in a broad spectrum of patients with cardiovascular disease. J Am Coll Cardiol.

[8] Mullens W, Abrahams Z, Francis GS (2009). Importance of venous congestion for worsening of renal function in advanced decompensated heart failure. J Am Coll Cardiol.

[9] Gnanaraj J, Radhakrishnan J (2016). Cardio-renal syndrome. F1000Res.

[10] Ronco C, Cicoira M, McCullough PA (2012). Cardiorenal syndrome type 1: pathophysiological crosstalk leading to combined heart and kidney dysfunction in the setting of acutely decompensated heart failure. J Am Coll Cardiol.

[11] Cruz DN, Bagshaw SM (2010). Heart-kidney interaction: epidemiology of cardiorenal syndromes. Int J Nephrol.

[12] Bagshaw SM, Cruz DN, Aspromonte N (2010). Epidemiology of cardio-renal syndromes: workgroup statements from the 7th ADQI Consensus Conference. Nephrol Dial Transplant.

[13] Ahmed A, Rich MW, Sanders PW (2007). Chronic kidney disease associated mortality in diastolic versus systolic heart failure: a propensity matched study. Am J Cardiol.

[14] Scabbia EV, Scabbia L (2015). The cardio-renal syndrome (CRS). IJC Metab Endoc.

[15] Garg AX, Clark WF, Haynes RB, House AA (2002). Moderate renal insufficiency and the risk of cardiovascular mortality: results from the NHANES I. Kidney Int.

[16] Sarnak MJ, Coronado BE, Greene T, Wang SR, Kusek JW, Beck GJ, Levey AS (2002). Cardiovascular disease risk factors in chronic renal insufficiency. Clin Nephrol.

[17] Keith DS, Nichols GA, Gullion CM, Brown JB, Smith DH (2004). Longitudinal follow-up and outcomes among a population with chronic kidney disease in a large managed care organization. Arch Intern Med.

[18] McCullough PA, Li S, Jurkovitz CT (2008). Chronic kidney disease, prevalence of premature cardiovascular disease, and relationship to short-term mortality. Am Heart J.

[19] Tonelli M, Wiebe N, Culleton B (2006). Chronic kidney disease and mortality risk: a systematic review. J Am Soc Nephrol.

[20] Gimbrone MA Jr, Topper JN, Nagel T, Anderson KR, Garcia-Cardeña G (2000). Endothelial dysfunction, hemodynamic forces, and atherogenesis. Ann N Y Acad Sci.

[21] Braunwald E (2008). Biomarkers in heart failure. N Engl J Med.

[22] Burns WC, Thomas MC (2011). Angiotensin II and its role in tubular epithelial to mesenchymal transition associated with chronic kidney disease. Cells Tissues Organs.

[23] Kraut EJ, Chen S, Hubbard NE, Erickson KL, Wisner DH (1999). Tumor necrosis factor depresses myocardial contractility in endotoxemic swine. J Trauma.

[24] Damman K, Valente MA, van Veldhuisen DJ (2017). Plasma neutrophil gelatinase-associated lipocalin and predicting clinically relevant worsening renal function in acute heart failure. Int J Mol Sci.

[25] Burnett JC Jr, Knox FG (1980). Renal interstitial pressure and sodium excretion during renal vein constriction. Am J Physiol.

[26] Cicoira M, Bolger AP, Doehner W (2001). High tumour necrosis factor-alpha levels are associated with exercise intolerance and neurohormonal activation in chronic heart failure patients. Cytokine.

[27] Glance LG, Wissler R, Mukamel DB (2010). Perioperative outcomes among patients with the modified metabolic syndrome who are undergoing noncardiac surgery. Anesthesiology.

[28] Warnock DG, Muntner P, McCullough PA (2010). Kidney function, albuminuria, and all-cause mortality in the REGARDS (Reasons for Geographic and Racial Differences in Stroke) study. Am J Kidney Dis.

[29] Levey AS, Eckardt KU, Dorman NM (2020). Nomenclature for kidney function and disease: report of a Kidney Disease: Improving Global Outcomes (KDIGO) Consensus Conference. Kidney Int.

[30] McAlister FA, Ezekowitz J, Tarantini L (2012). Renal dysfunction in patients with heart failure with preserved versus reduced ejection fraction: impact of the new Chronic Kidney Disease-Epidemiology Collaboration Group formula. Circ Heart Fail.

[31] Vergaro G, Del Franco A, Giannoni A (2015). Galectin-3 and myocardial fibrosis in nonischemic dilated cardiomyopathy. Int J Cardiol.

[32] Iacoviello M, Aspromonte N, Leone M (2016). Galectin-3 Serum levels are independently associated with microalbuminuria in chronic heart failure outpatients. Res Cardiovasc Med.

[33] Newman DJ, Thakkar H, Edwards RG, Wilkie M, White T, Grubb AO, Price CP (1995). Serum cystatin C measured by automated immunoassay: a more sensitive marker of changes in GFR than serum creatinine. Kidney Int.

[34] Pinsino A, Mondellini GM, Royzman EA (2020). Cystatin C- versus creatinine-based assessment of renal function and prediction of early outcomes among patients with a left ventricular assist device. Circ Heart Fail.

[35] Singh D, Whooley MA, Ix JH, Ali S, Shlipak MG (2007). Association of cystatin C and estimated GFR with inflammatory biomarkers: the Heart and Soul Study. Nephrol Dial Transplant.

[36] Nozawa Y, Sato H, Wakamatsu A (2018). Utility of estimated glomerular filtration rate using cystatin C and its interpretation in patients with rheumatoid arthritis under glucocorticoid therapy. Clin Chim Acta.

[37] Sharma A, Mucino MJ, Ronco C (2014). Renal functional reserve and renal recovery after acute kidney injury. Nephron Clin Pract.

[38] Comper WD, Hilliard LM, Nikolic-Paterson DJ, Russo LM (2008). Disease-dependent mechanisms of albuminuria. Am J Physiol Renal Physiol.

[39] Miller WG, Bruns DE, Hortin GL (2010). [Current issues in measurement and reporting of urinary albumin excretion]. Ann Biol Clin (Paris).

[40] Bazzi C, Petrini C, Rizza V, Arrigo G, Napodano P, Paparella M, D'Amico G (2002). Urinary N-acetyl-beta-glucosaminidase excretion is a marker of tubular cell dysfunction and a predictor of outcome in primary glomerulonephritis. Nephrol Dial Transplant.

[41] Robles NR, Lopez Gomez J, Garcia Pino G, Valladares J, Hernandez Gallego R, Cerezo I (2021). Alpha-1-microglobulin: Prognostic value in chronic kidney disease. Med Clin (Barc).

[42] George JA, Gounden V (2019). Novel glomerular filtration markers. Adv Clin Chem.

[43] Devuyst O, Olinger E, Rampoldi L (2017). Uromodulin: from physiology to rare and complex kidney disorders. Nat Rev Nephrol.

[44] Iacoviello M, Di Serio F, Rizzo C (2019). Association between high Gal-3 serum levels and worsening of renal function in chronic heart failure outpatients. Biomark Med.

[45] de Boer RA, Lok DJ, Jaarsma T, van der Meer P, Voors AA, Hillege HL, van Veldhuisen DJ (2011). Predictive value of plasma galectin-3 levels in heart failure with reduced and preserved ejection fraction. Ann Med.

[46] Grande D, Leone M, Rizzo C (2017). A multiparametric approach based on NT-proBNP, ST2, and galectin3 for stratifying one year prognosis of chronic heart failure outpatients. J Cardiovasc Dev Dis.

[47] Rebholz CM, Selvin E, Liang M (2018). Plasma galectin-3 levels are associated with the risk of incident chronic kidney disease. Kidney Int.

[48] Suthahar N, Meijers WC, Silljé HH, Ho JE, Liu FT, de Boer RA (2018). Galectin-3 activation and inhibition in heart failure and cardiovascular disease: an update. Theranostics.

[49] Schmidt-Ott KM, Mori K, Li JY, Kalandadze A, Cohen DJ, Devarajan P, Barasch J (2007). Dual action of neutrophil gelatinase-associated lipocalin. J Am Soc Nephrol.

[50] Mishra J, Dent C, Tarabishi R (2005). Neutrophil gelatinase-associated lipocalin (NGAL) as a biomarker for acute renal injury after cardiac surgery. Lancet.

[51] Murray PT, Wettersten N, van Veldhuisen DJ (2019). Utility of urine neutrophil gelatinase-associated lipocalin for worsening renal function during hospitalization for acute heart failure: primary findings of the urine N-gal Acute Kidney Injury N-gal Evaluation of Symptomatic Heart Failure Study (AKINESIS). J Card Fail.

[52] Noiri E, Doi K, Negishi K (2009). Urinary fatty acid-binding protein 1: an early predictive biomarker of kidney injury. Am J Physiol Renal Physiol.

[53] Price PM, Safirstein RL, Megyesi J (2009). The cell cycle and acute kidney injury. Kidney Int.

[54] Yang QH, Liu DW, Long Y, Liu HZ, Chai WZ, Wang XT (2009). Acute renal failure during sepsis: potential role of cell cycle regulation. J Infect.

[55] Bihorac A, Chawla LS, Shaw AD (2014). Validation of cell-cycle arrest biomarkers for acute kidney injury using clinical adjudication. Am J Respir Crit Care Med.

[56] Lekawanvijit S, Krum H (2014). Cardiorenal syndrome: acute kidney injury secondary to cardiovascular disease and role of protein-bound uraemic toxins. J Physiol.

[57] Meert N, Schepers E, De Smet R (2007). Inconsistency of reported uremic toxin concentrations. Artif Organs.

[58] Taguchi K, Fukami K, Elias BC, Brooks CR (2021). Dysbiosis related advanced glycation end products and trimethylamine N-oxide in chronic kidney disease. Toxins (Basel).

[59] Savira F, Cao L, Wang I (2017). Apoptosis signal-regulating kinase 1 inhibition attenuates cardiac hypertrophy and cardiorenal fibrosis induced by uremic toxins: Implications for cardiorenal syndrome. PLoS One.

[60] Miyazaki T, Ise M, Seo H, Niwa T (1997). Indoxyl sulfate increases the gene expressions of TGF-beta 1, TIMP-1 and pro-alpha 1(I) collagen in uremic rat kidneys. Kidney Int Suppl.

[61] Lin CJ, Wu V, Wu PC, Wu CJ (2015). Meta-analysis of the associations of p-cresyl sulfate (PCS) and indoxyl sulfate (IS) with cardiovascular events and all-cause mortality in patients with chronic renal failure. PLoS One.

[62] Felker GM, Lee KL, Bull DA (2011). Diuretic strategies in patients with acute decompensated heart failure. N Engl J Med.

[63] Testani JM, Kimmel SE, Dries DL, Coca SG (2011). Prognostic importance of early worsening renal function after initiation of angiotensin-converting enzyme inhibitor therapy in patients with cardiac dysfunction. Circ Heart Fail.

[64] Damman K, Voors AA, Navis G, van Veldhuisen DJ, Hillege HL (2012). Current and novel renal biomarkers in heart failure. Heart Fail Rev.

[65] VanVliet AA, Donker AJ, Nauta JJ (1993). Spironolactone in congestive heart failure refractory to high-dose loop diuretic and low-dose angiotensin-converting enzyme inhibitor. Am J Cardiol.

[66] van der Vliet A, O'Neill CA, Cross CE, Koostra JM, Volz WG, Halliwell B, Louie S (1999). Determination of low-molecular-mass antioxidant concentrations in human respiratory tract lining fluids. Am J Physiol.

[67] Collins SP, Hart KW, Lindsell CJ (2012). Elevated urinary neutrophil gelatinase-associated lipocalcin after acute heart failure treatment is associated with worsening renal function and adverse events. Eur J Heart Fail.

[68] Dormans TP, Gerlag PG (1996). Combination of high-dose furosemide and hydrochlorothiazide in the treatment of refractory congestive heart failure. Eur Heart J.

[69] Yusuf S, Pitt B, Davis CE, Hood WB, Cohn JN (1991). Effect of enalapril on survival in patients with reduced left ventricular ejection fractions and congestive heart failure. N Engl J Med.

[70] Go AS, Chertow GM, Fan D, McCulloch CE, Hsu CY (2004). Chronic kidney disease and the risks of death, cardiovascular events, and hospitalization. N Engl J Med.

[71] Heywood JT, Fonarow GC, Costanzo MR, Mathur VS, Wigneswaran JR, Wynne J (2007). High prevalence of renal dysfunction and its impact on outcome in 118,465 patients hospitalized with acute decompensated heart failure: a report from the ADHERE database. J Card Fail.

[72] Liangos O, Perianayagam MC, Vaidya VS (2007). Urinary N-acetyl-beta-(D)-glucosaminidase activity and kidney injury molecule-1 level are associated with adverse outcomes in acute renal failure. J Am Soc Nephrol.

[73] Masson S, Latini R, Milani V (2010). Prevalence and prognostic value of elevated urinary albumin excretion in patients with chronic heart failure: data from the GISSI-Heart Failure trial. Circ Heart Fail.

[74] Pinsino A, Mondellini GM, Royzman EA (2020). Cystatin C- versus creatinine-based assessment of renal function and prediction of early outcomes among patients with a left ventricular assist device. Circ Heart Fail.

[75] Ronco C, McCullough P, Anker SD (2010). Cardio-renal syndromes: report from the consensus conference of the acute dialysis quality initiative. Eur Heart J.

[76] Valente MA, Hillege HL, Navis G, Voors AA, Dunselman PH, van Veldhuisen DJ, Damman K (2014). The chronic kidney disease epidemiology collaboration equation outperforms the modification of diet in renal disease equation for estimating glomerular filtration rate in chronic systolic heart failure. Eur J Heart Fail.

[77] Vergaro G, Iacoviello M (2018). Is there a "renal paradox" in chronic heart failure?. Int J Cardiol.

[78] Kirklin JK, Naftel DC, Kormos RL (2013). Quantifying the effect of cardiorenal syndrome on mortality after left ventricular assist device implant. J Heart Lung Transplant.

